# Health-related quality of life and its associated factors among outpatients with heart failure: a cross-sectional study

**DOI:** 10.1186/s12955-023-02142-w

**Published:** 2023-07-13

**Authors:** Anan S. Jarab, Hanan W. Hamam, Walid A. Al-Qerem, Shrouq R. Abu Heshmeh, Tareq L. Mukattash, Eman A. Alefishat

**Affiliations:** 1 Department of Clinical Pharmacy, Faculty of Pharmacy, Jordan University of Science and Technology, P.O. Box 3030, Irbid, 22110 Jordan; 2 College of Pharmacy, Al Ain University, Abu Dhabi, UAE; 3 Department of Pharmacy, Faculty of Pharmacy, Al-Zaytoonah University of Jordan, P.O. Box 130, Amman, 11733 Jordan; 4 Department of Pharmacology, College of Medicine and Health Science, Khalifa University of Science and Technology, Abu Dhabi, 127788 United Arab Emirates; 5 Department of Biopharmaceutics and Clinical Pharmacy, Faculty of Pharmacy, The University of Jordan, Amman, 11942 Jordan; 6 Center For Biotechnology, Khalifa University of Science and Technology, Abu Dhabi, 127788 United Arab Emirates

**Keywords:** Heart failure, Health-related quality of life, Minnesota living with heart failure questionnaire, Intervention, Jordan

## Abstract

**Background:**

Heart Failure (HF) is a chronic disease associated with life-limiting symptoms that could negatively impact patients’ health-related quality of life (HRQOL). This study aimed to evaluate HRQOL and explore the factors associated with poor HRQOL among patients with HF in Jordan.

**Methods:**

This cross-sectional study used the validated Arabic version of the Minnesota Living with Heart Failure Questionnaire to assess HRQOL in outpatients with HF visiting cardiology clinics at two public hospitals in Jordan. Variables were collected from medical records and custom-designed questionnaires, including socio-demographics, biomedical variables, and disease and medication characteristics. Ordinal regression analysis was used to explore variables associated with poor HRQOL among HF patients.

**Results:**

Ordinal regression analysis showed that the number of HF medications (P < 0.05) and not taking a loop diuretic (P < 0.05) significantly increased HRQOL, while the number of other chronic diseases (P < 0.05), stage III/IV of HF (P < 0.01), low monthly income (P < 0.05), and being unsatisfied with the prescribed medications (P < 0.05) significantly decreased HRQOL of HF patients.

**Conclusions:**

Although the current study demonstrated low HRQOL among patients with HF in Jordan, HRQOL has a considerable opportunity for improvement in those patients. Variables identified in the present study, including low monthly income, higher New York Heart Association (NYHA) classes, a higher number of comorbidities, and/or taking a loop diuretic, should be considered in future intervention programs, aiming to improve HRQOL in patients with HF.

## Background

Heart failure (HF) is a chronic, progressive disease characterized by the inability of the myocardium to pump enough blood to meet the body’s needs [1]. It has been an increasing public health problem in developing and developed countries [2], with around 64.3 million people having HF worldwide [3]. In National Health and Nutrition Examination Survey data, an estimated 6.2 million American adults aged 20 years and older had HF between 2013 and 2016 compared with an estimated 5.7 million between 2009 and 2012 [4]. Furthermore, the prevalence of HF is expected to rise by 46% from 2012 to 2030, resulting in more than 8 million people aged 18 years and older having the disease in the United States [5]. Although effective therapeutic interventions are available, the mortality rates in patients with HF remained high [6]. According to recent statistics in Jordan, cardiovascular diseases (CVDs), including HF, contributed to about 37% of all deaths in the country [7]. HF has a significant burden on the healthcare system, and its management takes approximately 1–2% of healthcare expenses [8, 9]. In 2012, the total cost of HF was estimated to be $30.7 billion, of which 68% was attributable to direct medical costs, and it was suggested that by 2030, the total cost of HF will increase almost by 127% to reach 69.7 billion dollars [5].

Serious complications are known to be associated with HF progression, such as hospitalization, arrhythmias, and, ultimately, death [10]. Patients with HF experience various life-limiting symptoms such as dyspnea, fatigue, edema, sleeping difficulties, chest pain, and depression [11]. Moreover, they suffer from different and significant physical, psychological, and social burdens, resulting in poor health-related quality of life (HRQOL) [12, 13]. Previously published studies indicated that HRQOL in patients with HF is greatly impaired when compared with healthy populations as well as those with other chronic diseases [14–16]. HRQOL refers to how well a person functions in daily activities and perceives well-being in the physical, psychological, and health-related social aspects [17]. It has been recognized as an important clinical indicator that predicts mortality among patients with HF as well as the effectiveness of the introduced health services [18, 19]. Several factors have been identified that influence HRQOL among patients with HF [20–22]. The diversity of these factors, in addition to the negative impact of HF on HRQOL, necessitates the implementation of further studies to reveal the true predictors of poor HRQOL among HF patients. In Jordan, although several factors have been linked to poor HRQOL in other disease settings [23, 24], no studies have explored HRQOL among HF patients. Therefore, this study aimed to evaluate HRQOL and explore the factors associated with poor HRQOL among patients with HF in Jordan.

## Methods

### Study design and settings

The current cross-sectional study was conducted on patients with HF attending the outpatient cardiology clinics at King Abdullah University Hospital (KAUH) and Al Bashir Hospital in Jordan in the period from August 2021 through April 2022.

### Sampling

All patients who were aged 18 years or older, had a confirmed diagnosis of HF for at least six months, had a cardiologist’s assignment of New York Heart Association (NYHA) classification, were taking at least one HF medication, and agreed to sign a consent form were included in the study. Patients with acute decompensation of HF or an active listing for heart transplantation and patients with cognitive impairment were excluded from the study.

Eligible patients were informed that participation is voluntary. They have the right to withdraw from the study at any time, and their medical care and treatment will not be affected by their participation. They were also informed that the collected data would only be used for research purposes and would be kept in the Principal Investigator’s office to ensure confidentiality. The interview took approximately 10–15 min to be completed.

### Study instruments

During outpatient clinics visits, the research pharmacist used a custom-designed questionnaire to collect socio-demographic data such as age, gender, body mass index (BMI), marital status, place of residency, living arrangements, education, occupation status, monthly income, smoking status, physical activity, and family history of cardiovascular diseases. The researcher used the medical records to collect information about the duration of HF, NYHA heart failure classification, the presence and number of other comorbid diseases, and medication-related information. The collected information also included different biomedical and laboratory data such as low-density lipoprotein (LDL), high-density lipoprotein (HDL), triglycerides, total cholesterol, glycosylated haemoglobin (HbA1c), random blood glucose, systolic blood pressure (SBP), diastolic blood pressure (DBP), ejection fraction (EF%), serum creatinine (SCr), white blood cells (WBC), red blood cells (RBC) and haemoglobin (Hb).

### The 4-item medication adherence scale

Patients’ willingness to take their medications as prescribed was measured with this simple, validated, 4-item survey, illustrating a variety of reasons for medication non-adherence, such as forgetting, carelessness, stopping the medication when feeling better, and stopping the medication when feeling worse [25]. One score was given for each ‘yes’ response, and each ‘no’ response was given a score of zero. The scores ranged from 0 to 4. According to the Morisky classification, adherence was divided into three groups: high for those scoring zero, moderate for those scoring one or two, and low for those scoring three or four. For the purpose of this study, we used a validated Arabic version of this tool [26].

### The Minnesota living with heart failure questionnaire (MLHFQ)

This questionnaire is one of the most widely used tools in clinical settings for measuring HRQOL in HF patients, with proven reliability and validity [27]. It is short, easily understood by ill and elderly individuals, and easy to score.

MLHFQ contains questions that measure the effect of common physical symptoms on HF, like shortness of breath, fatigue, peripheral edema, and difficulty sleeping, as well as psychological symptoms, such as anxiety and depression. Furthermore, the impact of HF on physical/social functions, including walking, climbing stairs, household work, need to rest, earning a living, going away from home, doing activities with family or friends, entertainment, and sexual activities, eating behaviour, memory, loss of self-control, and being a burden to others were incorporated into the measure. Questions about medications’ side effects, hospital stays, and costs of care were also included to help measure the overall impact of treatment on quality of life. The patients answered those 21 questions according to the last month. Possible answers range from 0 (no effect) to 5 (very much), with higher scores indicating poorer HRQOL. The final score is the sum of the points obtained from the 21 questions (ranging from 0 to 105). Scores below 24 indicated good HRQOL, scores from 24 to 45 indicated moderate HRQOL, while scores higher than 45 indicated poor HRQOL [28]. A validated and reliable Arabic version of MLHFQ was used in this study [29].

All parts of the survey were completely self-reported, and patients who faced difficulty-completing questionnaires had the questions read to them without giving any interpretation.

### Data analysis

Using the Statistical Package for the Social Sciences (SPSS version 26 from IBM), descriptive and analytical statistics were done. Continuous variables were presented using descriptive analyses in terms of means (standard deviations) and the categorical variables in terms of frequencies (percentages). The differences between patients about HRQOL were examined using the independent sample t-test and the Mann–Whitney U-test for normally and non-normally distributed continuous variables, respectively. Pearson Chi-square test was conducted to discover the association between the categorical variables and HRQOL. Variables with a *p-value* < 0.05 at the univariate analysis were fitted into the multinomial logistic regression to discover significant and independent predictors of HRQOL.

## Results

A total of 427 out of 550 HF patients (response rate 77.6%) were included in the study. As shown in Table 1, the participants’ mean age was 62.58 ± 11.7. The majority of the participants were males (63.7%), married (94.4%), living with their families (94.6%), living in the city (66.8%), did not complete their education (69.4%), unemployed or retired (77.6%), had a monthly income of less than 500 JD (67.1%), and non-smokers (69.9%). More than half of the patients had no family history of cardiovascular diseases (51.2%). More information about the demographic characteristics of the study participants is presented in Table 1.

**Table 1:** Demographic characteristics of the study participants (n = 427)

Characteristic	Mean (± SD ^a^)		n (%)
**Age**	62.58 (**±** 11.7)		
**Gender**			
Male			272 (63.7)
Female			155 (36.3)
**Body Mass Index (kg/m** ^**2**^ **)**			
Normal (≤ 24.9)			90 (21.8)
Overweight (25–29.9)			161 (39)
Obese (≥ 30)			162 (39.2)
**Marital status**			
Married			404 (94.4)
Single/ Divorced/ Widowed			24 (5.6)
**Living conditions**			
Living alone			23 (5.4)
Living with family/ other			405 (94.6)
**Residency**			
City			286 (66.8)
Countryside			142 (33.2)
**Education**			
University/ Collage			131 (30.6)
Less than collage			297 (69.4)
**Working status**			
Employed			96 (22.4)
Unemployed/ Retired			332 (77.6)
**Monthly income**			
Less than 500 JD ^**b**^			287 (67.1)
500–1000 JD ^**b**^			141 (32.9)
**Smoking status**			
Current Smoker			129 (30.1)
Non-smoker			299 (69.9)
**Physical activity**			
Active			51 (11.9)
Not active			377 (88.1)
**Family history of CVD** ^**c**^			
Yes			209 (48.8)
No			219 (51.2)

^**a**^ Standard deviation; ^**b**^ JD: Jordanian Dinar; ^c^ cardiovascular diseases

As shown in Table 2, HF duration in years had a mean of 6.4 (± 6.16). A number of other comorbidities had a mean of 3 (± 1), and the most common comorbid conditions among those patients were hypertension (77.1%), diabetes mellitus (61.2%), and ischemic heart disease (64.3%). The majority of patients were classified as group III/IV according to NYHA classification (69.6%). HF patients in our study were taking 8 (± 3) medications on average, and the number of HF medications mean was 3 (± 1). The most commonly prescribed HF medications were BBs (88.6%), CCBs (70.8%), loop diuretics (67.5%), and ACEIs/ ARBs (66.8%). The majority of the patients were satisfied with their medications (77.3%), had no fears of the side effects of the medications (71.3%), did not experience any side effects (68.0%), reported moderate adherence to HF medications (84.2%), followed by low (8.8%) and high medication adherence (7.0%).

**Table 2:** Disease and medication characteristics of the study participants (n = 427)

Variables	n (%)	Mean (SD ^a^)
**Disease related**		
Duration of heart failure (years)		6.41 (± 6.16)
Number of other chronic diseases		3 (± 1)
NYHA ^b^ classification		
I/II	130 (30.4)	
III/IV	298 (69.6)	
**Type of comorbidities**		
Hypertension	330 (77.1)	
Diabetes Mellitus	262 (61.2)	
Ischemic Heart Diseases	275 (64.3)	
Dyslipidemia	142 (33.2)	
Chronic Kidney Disease	56 (13.1)	
Thyroid Dysfunction	36 (8.4)	
**Heart failure medications**		
Aldosterone Antagonist	81 (18.9)	
Digoxin	46 (10.7)	
ACEIs ^c^/ARBs ^d^	286 (66.8)	
Beta blocker	379 (88.6)	
Loop diuretic	289 (67.5)	
Vasodilator	79 (18.5)	
Calcium Channel Blocker	81 (18.9)	
Ivabradine	20 (4.7)	
Valsartan/ Sacubitril	31 (7.2)	
**Other medications**		
Anticoagulant	148 (34.6)	
Statin	303 (70.8)	
Number of Medications		8 (± 3)
Number of heart failure medications		3 (± 1)
**Medications frequency**		
Once	78 (18.2)	
Twice	249 (58.2)	
Thrice or more	101 (23.6)	
**Fear of medications’ side effects**		
No	305 (71.3)	
Yes	123 (28.7)	
**Medications satisfaction**		
No	97 (22.7)	
Yes	331 (77.3)	
**Experiencing side effects**		
No	291 (68)	
Yes	137 (32)	

^**a**^ Standard deviation; ^**b**^ The New York Heart Association Classification; ^**c**^ Angiotensin-converting enzyme inhibitors; ^**d**^ Angiotensin-receptor blockers

As shown in Table 3, the means of blood pressure readings were 129 (± 22) for SBP and 76 (± 22) for DBP. Most of the study participants had HF with reduced ejection fraction (HFrEF) Other biomedical variables are presented in Table 3.

**Table 3:** Biomedical tests of the study participants (n = 427)

Variable	Mean (± SD)
HbA1c (%)	7.28 (± 1.97)
TGs (mmol/L)	1.96 (± 1.169)
TC (mmol/L)	4.18 (± 1.288)
LDL (mmol/L)	2.73 (± 5.15)
HDL (mmol/L)	1.08 (± 1.795)
Non-HDL Cholesterol (mmol/L)	2.86 (± 2.21)
SCr (micmol/L)	108.15 (± 78.43)
Hb (g/dL)	16.61 (± 74.85)
WBCs count (*10^9^/L)	8.84 (± 5.37)
RBCs count (*10^9^/L)	4.68 (± 0.92)
Ejection Fraction	
Reduced (≤ 40%)	157 (47.6%)
Mildly reduced (41–49%)	78 (23.6%)
Preserved (≥ 50%)	95 (28.8%)

SD: Standard deviation; Hb: Hemoglobin; TGs: Triglycerides; TC: Total cholesterol; LDL: Low-density lipoproteins; HDL: High-density lipoproteins, SCr: Serum creatinine; WBCs: White blood cells; RBCs: Red blood cells; SBP: Systolic blood pressure; DBP: Diastolic blood pressure

According to the 4-item Medication Adherence Scale, the majority of the patients showed moderate adherence to HF medications (83.6%), 8.9% showed low adherence, while only 7.5% had high medication adherence. According to MLHFQ, nearly half of the patients were in the poor HRQOL group (47.2%).

Univariate analysis showed that age, BMI, gender, educational level, job status, monthly income, NYHA classification, number of comorbidities other than HF, hypertension, having diabetes, medications satisfaction, total number of medications, number of HF medications, taking loop diuretic, fear of and experiencing side effects, SCr, HbA1c, Hb, and RBC were significantly associated with HRQOL. Ordinal regression analysis was conducted to reveal the variables that were significantly associated with HRQOL in HF patients. The variables that were significantly associated with HRQOL in the univariate analysis were included in the model as independent variables. Results of the ordinal regression analysis (Table 4) showed that the number of HF medications (P < 0.05) and not taking loop diuretic (P < 0.05) were significantly associated with improved HRQOL. On the other hand, number of other chronic diseases (P < 0.0.05), class III/IV of HF according to NYHA (P < 0.01), low monthly income (P < 0.05), and being unsatisfied with the prescribed medications (P < 0.05) significantly decreased HRQOL.

**Table 4:** Ordinal regression analysis of the variables associated with HRQOL

Variable			Coefficient estimate	(95% CI)		P
**Number of heart failure medications**			0.277	(0.037–0.518)		0.024*
**Number of other chronic diseases**			-0.303	(-0.555)-0.051))		0.018*
**NYHA** ^**a**^ **classification**						
		I/II	Reference			< 0.001**
		III/IV	-2.288	(-2.832)-1.744)		
**Monthly income**						
		more than 500 JD	Reference			0.017*
		less than 500 JD	-0.722		(-1.317)-0.127)	
**Taking a loop diuretic**						
		Yes	Reference			0.030*
		No	0.595	(0.058–1.133)		
**Medications satisfaction**						
		Yes	Reference			0.025*
		No	-0.604	(-1.131-0.077)		

^a^ New York Heart Association

* Significant at P < 0.05;** Significant at P < 0.01

## Discussion

Quality of life for patients living with HF is not less important than prolonged survival rates; it also deserves attention [30]. Hobbs et al. found that HF patients had a significant impairment in all aspects of their quality of life, and that physical health burden associated with HF was significantly higher than that associated with other chronic diseases [6]. An earlier Jordanian study that assessed HRQOL from HF patients’ perspectives revealed that HF had a negative impact on patient’s HRQOL, especially in physical domain [31]. To the best of our knowledge, this study is the first one to address HRQOL and its associated factors among HF patients in Jordan.

According to the current study results, approximately half of the participants (47.2%) had poor HRQOL. Consistent results were reported in earlier studies [32, 33] which used Minnesota Living with Heart Failure Questionnaire (MLHFQ) and found that half of the participating HF patients had poor HRQOL. Another study which was conducted among Arab patients with HF using the Short Form 36 (SF-36) found that their HRQOL was impaired, and patients reported significant disruptive pain and fatigue, interference with social activities, impaired psychological status, and limitations performing usual activities [34]. Another study which used the SF-36 questionnaire reflected poor HRQOL among HF patients in Greece [35].

The current study results showed a significant relationship between lower income and poor HRQOL. Low income has been reported as a predictor of poor HRQOL in term of physical aspect and in terms of both physical and emotional aspects in patients with HF [36, 37]. Other studies reported that lower socioeconomic status had a negative impact on the overall scores of HRQOL among patients with HF [38, 39]. Low income may reduce patients’ desire to make required clinic visits or adhere to their medication, which would negatively impact the health status and hence HRQOL of these patients [12].

The current study results revealed a negative relationship between number of comorbidities and HRQOL, which was similar to the findings reported in several other studies [40–43]. Comorbidities amongst HF patients, such as diabetes, peripheral vascular disease, and cerebrovascular disease are responsible for frequent hospitalization, poor performance in activities, and increased health care expenditures [11, 39, 43] which may play a role in reducing HRQOL in these patients.

A negative impact of higher NYHA classification on HRQOL was observed in the current study. Consistent findings were reported in several other studies conducted among patients with HF, which found that patients with higher classes of NYHA had significantly poorer HRQOL than those with lower NYHA classification [2, 36, 38, 44]. These findings could be justified by the fact that NYHA classification depends on symptoms and physical limitations experienced by HF patients, which could reflect their HRQOL [40]. Logically, patients with higher NYHA classification had worse symptoms, greater physical limitation, and thus poorer HRQOL.

Although loop diuretics therapy are the mainstay of HF management and has been used for decades [45], our study results showed that patients who were not taking loop diuretics had better HRQOL than those who were, which was consistent with previous studies findings that found a negative impact of taking diuretics on HRQOL [46, 47]. Domanski et al. reported that patients receiving loop diuretics usually have more advanced and symptomatic stages that may reflect more severe disease and poorer HRQOL [48]. Furthermore, a study constructed by Paran et al. on hypertensive patients found that patients on diuretics experienced more gastrointestinal symptoms, weakness, and sex related problems, and thus, poorer HRQOL, and that cessation of diuretics treatment for at least 9 months improved some aspects of patients’ HRQOL [49].

Contradictory to the existing literature report, a positive association between the number of HF medications and HRQOL was found in the present study [36, 50]. A possible explanation for this finding may be that physicians prescribe additional medications to better control symptoms of HF, which in term lead to improved HRQOL [20].

The current study showed a positive association between treatment satisfaction and HRQOL. Earlier Studies on hypertensive and diabetic patients have confirmed this association [51, 52]. Another study conducted among patients with multiple sclerosis (MS) reported that treatment satisfaction regarding side effects and effectiveness was an independent predictor of physical and mental HRQOL in patients with MS [53]. Patients’ satisfaction with their medications or the services they receive has been shown to guide medication adherence, treatment success, and hence HRQOL improvement [54, 55]. According to Jneid et al., higher levels of physician trust were associated with better treatment satisfaction and adherence [51]. Therefore, to achieve HF optimum management, prescribers must consider trust creation, not only by providing the effective treatment but also through effective communication and establishing a therapeutic relationship along with patient engagement in treatment decision making.

This study has some limitations; social desirability bias associated with using self-reporting for assessing HRQOL may have affected the accuracy of patients’ responses. However, this study used a disease specific instrument and a sample size higher than the required, which provided a robust findings and unique insights into the predictors of poor HRQOL among Jordanian patients with HF.

## Conclusions

The current study is the first to explore factors associated with HRQOL among patients with HF in Jordan. A margin for improvement in HRQOL among patients with HF in Jordan was demonstrated in this study. However, patients with low monthly income, higher NYHA classes, a higher number of comorbidities, and those on loop diuretics need to be in future pharmaceutical intervention programs in order to improve HRQOL in patients with HF.

## Data Availability

The datasets used and/or analyzed during the present study are available from the corresponding author on reasonable request.

## List of Abbreviations

ACEI: Angiotensin converting enzyme inhibitor
ARB: Angiotensin receptor blocker
BB: β-blockers
CCB: Calcium channel blocker
CVD: Cardiovascular disease
DBP: Diastolic blood pressure
EF: Ejection fraction
Hb: Haemoglobin
HDL: High-density lipoprotein
HF: Heart failure
HRQOL: Health related quality of life
JD: Jordanian dinar
KAUH: King Abdullah University Hospital
LDL: low-density lipoprotein
MLHFQ: Minnesota Living with Heart Failure Questionnaire
NYHA: New York Heart Association
RBC: Red blood cell
SBP: Systolic blood pressure
SCr: Serum creatinine
SPSS: Statistical Package for the Social Sciences
WBC: White blood cell
